# Is the call to abandon *p*-values the red herring of the replicability crisis?

**DOI:** 10.3389/fpsyg.2015.00245

**Published:** 2015-03-06

**Authors:** Victoria Savalei, Elizabeth Dunn

**Affiliations:** Department of Psychology, University of British ColumbiaVancouver, BC, Canada

**Keywords:** null hypothesis significance testing (NHST), *p*-values, confidence intervals (CIs), Bayes factors, crisis of replicability

## Introduction

In a recent article, Cumming (2014) called for two major changes to how psychologists conduct research. The first suggested change—encouraging transparency and replication—is clearly worthwhile, but we question the wisdom of the second suggested change: abandoning *p*-values in favor of reporting confidence intervals (CIs) only in all psychological research reports. This article has three goals. First, we correct the false impression created by Cumming that the debate about the usefulness of NHST has been won by its critics. Second, we take issue with the implied connection between the use of NHST and the current crisis of replicability in psychology. Third, while we agree with other critics of Cumming (2014) that hypothesis testing is an important part of science (Morey et al., 2014), we express skepticism that alternative hypothesis testing frameworks, such as Bayes factors, are a solution to the replicability crisis. Poor methodological practices can compromise the validity of Bayesian and classic statistical analyses alike. When it comes to choosing between competing statistical approaches, we highlight the value of applying the same standards of evidence that psychologists demand in choosing between competing substantive hypotheses.

## Has the NHST debate been settled?

Cumming (2014) claims that “very few defenses of NHST have been attempted” (p. 11). In a section titled “Defenses of NHST,” he summarizes a single book chapter by Schmidt and Hunter (1997), which in fact is not a defense but another critique, listing and “refuting” arguments for continued use of NHST^1^. Thus, graduate students and others who are new to the field might understandably be left with the impression that the debate over NHST has been handily won by its critics, with little dissent. This impression is wrong. Indeed, the book that published Schmidt and Hunter's (1997) chapter (Harlow et al., 1997) included several defenses (e.g., Abelson, 1997; Mulaik et al., 1997), and many contributions with more nuanced and varied positions (e.g., Harris, 1997; Reichardt and Gollob, 1997). Defenses have also appeared in the field's leading peer-reviewed journals, including *American Psychologist* (Krueger, 2001, with commentaries) and APA's quantitative psychology journal *Psychological Methods* (Frick, 1996; Cortina and Dunlap, 1997; Nickerson, 2000). Nickerson (2000) provided a particularly careful and thoughtful review of the entire debate and concluded “that NHST is easily misunderstood and misused but that when applied with good judgment it can be an effective aid to the interpretation of experimental data” (abstract). Perhaps the most famous critique of the use of NHST in psychology (Cohen, 1994), published in the *American Psychologist*, has seen several defending commentaries (Baril and Cannon, 1995; Frick, 1995; Parker, 1995), plus a lengthier retort (Hagen, 1997). We do not believe that the debate about the appropriate use of NHST in psychology has been decisively settled. Further, the strong NHST-bashing rhetoric common on the “reformers” side of the debate may prevent many substantive researchers from feeling that they can voice legitimate reservations about abandoning the use of *p*-values.

## Is the replicability crisis caused by NHST?

Cumming (2014) connects the current crisis in the field (e.g., Pashler and Wagenmakers, 2012) to “the severe flaws of null-hypothesis significance testing (NHST).” In our opinion, the reliance of psychologists on NHST is a red herring in the debates about the replicability crisis (see also Krueger, 2001). Cumming cites Ioannidis (2005) to draw the connection between NHST and the replicability crisis. Yet, Cumming does not explain how the fundamental problems articulated by Ioannidis (2005) could be resolved by abandoning NHST and focusing on CIs. Ioannidis (2005) described the intersecting problems that arise from running underpowered studies, conducting numerous statistical tests, and focusing only on the significant results. There is no evidence that replacing *p*-values with CIs will circumvent these problems^2^. After all, *p*-values and CIs are based on the same information, and are thus equivalently susceptible to “hacking.”

While Cumming warns that using CIs in the same way we use NHST (to reach a binary decision) would be a mistake and advocates not focusing on whether a CI includes zero, it is difficult to imagine researchers and editors ignoring this salient information. In fact, we feel that all claims about the superiority of one statistical technique over another in terms of facilitating correct interpretation and reasoning should be supported by evidence, as we would demand of any other claim made within our discipline. The only experimental study evaluating whether presenting data in terms of CIs reduces binary thinking relative to NHST did not find this to be the case^3^ (Hoekstra et al., 2012; see also Poitevineau and Lecoutre, 2001). Another purported advantage of abolishing *p*-values is that using CIs may make it easier to detect common patterns across studies (e.g., Schmidt, 1996). However, a recent experiment found that presenting the results of multiple studies in terms of CIs rather than in NHST form did not improve meta-analytic thinking (Coulson et al., 2010)^4^. It has also been argued that CIs might help improve research practices by making low power more salient, because power is directly related to the width of the confidence interval. There is some evidence that presenting data in terms of CIs rather than *p*-values makes people less vulnerable to interpreting non-significant results in under-powered studies as support for the null hypothesis (Fidler and Loftus, 2009; Hoekstra et al., 2012). Unfortunately, our reading of this research also suggests that using CIs pushed many participants in the opposite direction, and they tended to interpret CIs that include zero as moderate evidence for the alternative hypothesis. It is worth debating which of these interpretations is more problematic, a judgment call that may depend on the nature of the research. Finally, existing data do not support the notion that CIs are more intuitive. Misinterpretations of the meaning of CIs are as widespread as misinterpretations of *p*-values^5^ (Belia et al., 2005; Hoekstra et al., 2014). Abolishing *p*-values and replacing them with CIs, thus, is not a panacea.

Successfully addressing the replicability crisis demands fundamental changes, such as running much larger studies (Button et al., 2013; Vankov et al., 2014), directly replicating past work (Nosek et al., 2012), publishing null results, avoiding questionable research practices that increase “researcher degrees of freedom” (Simmons et al., 2011; John et al., 2012), and practicing open science more broadly. To the extent that replacing *p*-values with CIs appears to be an easy, surface-level “solution” to the replicability crisis—while doing little to solve the problems that caused the crisis in the first place—this approach may actually distract attention away from deeper, more effective changes.

## Are Bayes factors the solution to the replicability crisis?

Bayes factors have gained some traction in psychology as an alternative hypothesis-testing framework (e.g., Rouder et al., 2009; Dienes, 2011; Kruschke, 2011). This approach may be logically superior in that Bayes factors directly address the relative evidence for the null hypothesis vs. the alternative. Another major advantage is that Bayes factors force researchers to articulate their hypotheses in terms of prior distributions on the effect sizes. A simple “H_1_: μ > 0” will no longer do the trick, and the answer to the question “Is my hypothesis supported by the data?” will depend on the exact form of that hypothesis. Decades ago, Meehl (1990) argued that such a development was needed to push the science of psychology forward.

In the wake of the replicability crisis, some have argued that switching to Bayesian hypothesis testing can help remedy the bias against publishing non-significant results because, unlike NHST, Bayes factors allow researchers to establish support for the null (Dienes, 2014). More evidence is needed, however, that the switch to Bayes factors will have this effect. To the extent that the real source of publication bias is the pressure felt by journal editors to publish novel, striking findings, the rate of publication of null results will not increase, even if those null results are strongly supported by a Bayesian analysis. Further, when it comes to questionable research practices, one can “b-hack” just as one can “p-hack” (Sanborn and Hills, 2014; Simonsohn, 2014; Yu et al., 2014). In fact, Bayes factors and the values of the classic *t*-test are directly related, given a set sample size and choice of prior (Rouder et al., 2009; Wetzels et al., 2011). Although some have argued that the options for “b-hacking” are more limited (e.g., Wagenmakers, 2007, in an online appendix; Dienes, 2014; Rouder, 2014), no statistical approach is immune to poor methodological practices.

Furthermore, as pointed out by Simmons et al. (2011), using Bayes factors further increases “researcher degrees of freedom,” creating another potential QRP, because researchers must select a prior—a subjective expectation about the most likely size of the effect—for their analyses. Although the choice of prior is often inconsequential (Rouder et al., 2009), different priors can lead to different conclusions. For example, in their critique of Bem's (2011) article on pre-cognition, Wagenmakers et al. (2011) have devoted much space to the reanalysis of the data using Bayes factors, and less to pointing out the exploratory flexibility of many of Bem's (2011) analyses. Bem's response to this critique (Bem et al., 2011) was *entirely* about the Bayesian analyses—debating the choice of prior for psi. Given that the publication of Bem's (2011) article was one of the factors that spurred the current crisis, this statistical debate may have been a red herring, distracting researchers from the much deeper concerns about QRP's.

## Conclusion

We agree with Cumming (2014) that raw effect sizes and the associated CIs should routinely be reported. We also believe that Bayes factors represent an intriguing alternative to hypothesis testing via NHST. But, at present we lack empirical evidence that encouraging researchers to abandon *p*-values will fundamentally change the credibility and replicability of psychological research in practice. In the face of crisis, researchers should return to their core, shared value by demanding rigorous empirical evidence before instituting major changes.

### Conflict of interest statement

The authors declare that the research was conducted in the absence of any commercial or financial relationships that could be construed as a potential conflict of interest.

## Footnotes

^1^See Krantz (1999) for a criticism of the faulty logic in this chapter.

^2^For instance, Ioannidis's (2005) main example (Box 1) is a hypothetical study with the goal to test whether any of the 100,000 gene polymorphisms are associated with susceptibility to schizophrenia, with the prior odds for any one polymorphism set to be 0.0001, and with the power of 60% to detect any one association. It is unclear how this intersection of problems, which plagues all exploratory research, can be solved with CIs.

^3^We recognize the irony of drawing a binary inference of no evidence from this study, but the authors also reach this conclusion (and they also present both CIs and *p*-values to support their conclusions).

^4^Participants were from three different fields with varying statistical practices. As Coulson et al. (2010) noted: “Confidence intervals have been routinely reported in medical journals since the mid-1980s, yet our MED (medical) respondents did not perform notably better than BN (behavioral neuroscience) and PSY (psychology) respondents” (p. 8).

^5^ In fact, Cumming (2014) himself gives some decidedly Bayesian interpretations of CIs.
